# Identification of Novel and Recurrent Disease-Causing Mutations in Retinal Dystrophies Using Whole Exome Sequencing (WES): Benefits and Limitations

**DOI:** 10.1371/journal.pone.0158692

**Published:** 2016-07-08

**Authors:** Amit Tiwari, Johannes Lemke, Janine Altmueller, Holger Thiele, Esther Glaus, Johannes Fleischhauer, Peter Nürnberg, John Neidhardt, Wolfgang Berger

**Affiliations:** 1 Institute of Medical Molecular Genetics, University of Zurich, Wagistrasse 12, CH-8952, Schlieren, Switzerland; 2 Cologne Center for Genomics (CCG), University of Cologne, Weyertal 115b, D-50931, Cologne, Germany; 3 Department of Ophthalmology, University Hospital Zurich, Frauenklinikstrasse 24, CH-8091, Zürich, Switzerland; 4 Center for Molecular Medicine Cologne (CMMC), University of Cologne, Robert-Koch Str. 21, D-50931, Cologne, Germany; 5 Cologne Excellence Cluster on Cellular Stress Responses in Aging-Associated Diseases (CECAD), University of Cologne, Joseph-Stelzmann-Str. 26, D-50931, Cologne, Germany; 6 Zurich Center for Integrative Human Physiology (ZIHP), University of Zurich, Winterthurerstrasse 190, CH-8057, Zurich, Switzerland; 7 Neuroscience Center Zurich (ZNZ), University and ETH Zurich, Winterthurerstrasse 190, CH-8057, Zurich, Switzerland; Hadassah-Hebrew University Medical Center, ISRAEL

## Abstract

Inherited retinal dystrophies (IRDs) are Mendelian diseases with tremendous genetic and phenotypic heterogeneity. Identification of the underlying genetic basis of these dystrophies is therefore challenging. In this study we employed whole exome sequencing (WES) in 11 families with IRDs and identified disease-causing variants in 8 of them. Sequence analysis of about 250 IRD-associated genes revealed 3 previously reported disease-associated variants in *RHO*, *BEST1* and *RP1*. We further identified 5 novel pathogenic variants in *RPGRIP1* (p.Ser964Profs*37), *PRPF8* (p.Tyr2334Leufs*51), *CDHR1* (p.Pro133Arg and c.439-17G>A) and *PRPF31* (p.Glu183_Met193dup). In addition to confirming the power of WES in genetic diagnosis of IRDs, we document challenges in data analysis and show cases where the underlying genetic causes of IRDs were missed by WES and required additional techniques. For example, the mutation c.439-17G>A in *CDHR1* would be rated unlikely applying the standard WES analysis. Only transcript analysis in patient fibroblasts confirmed the pathogenic nature of this variant that affected splicing of *CDHR1* by activating a cryptic splice-acceptor site. In another example, a 33-base pair duplication in *PRPF31* missed by WES could be identified only via targeted analysis by Sanger sequencing. We discuss the advantages and challenges of using WES to identify mutations in heterogeneous diseases like IRDs.

## Introduction

Inherited retinal dystrophies (IRDs) are a group of rare but highly heterogeneous genetic disorders characterized by an abnormal function or degeneration of specific cell types in the retina, as for example photoreceptors. Consequently, partial or complete vision loss is experienced by affected individuals. These diseases are heterogeneous, not only in terms of age of onset, severity and progression of the disease, but also in terms of their underlying genetics[1]. Currently, there are around 250 genes, mutations in which have been reported to cause various forms of retinal dystrophies. These mutations can be inherited in an autosomal recessive, dominant or X-linked manner. Based on cells that are affected first during disease-progression, these diseases are also classified as either rod-dominated (e.g. retinitis pigmentosa, RP) or cone-dominated (e.g., cone-rod dystrophy, CORD). Moreover, mutations in the same gene have been shown to lead to variable phenotypes, adding to the already existing complexity.

Whole exome sequencing (WES) is an efficient method to identify disease-causing mutations, particularly for monogenic inherited disorders such as IRDs[2–4]. Although fast and accurate, WES fails to identify disease-causing mutations in almost 35% of the cases (Tiwari et al, unpublished data). Possible reasons include (i) variants in genes not yet disease-associated, (ii) variants that lie within deep intronic regions and are therefore missed by the exome capture methods, or (iii) limitations of the employed method that prevent efficient identification of sequence alterations. Complementary methods, e.g. autozygosity mapping or whole genome sequencing may be considered to facilitate the identification of the disease-associated genetic alterations.

General diagnostic approaches, applied in majority of the genetic laboratories, include Sanger sequencing of most frequently disease-associated gene(s), followed by either panel or whole exome sequencing. In this study, the majority of cases were first screened by Sanger sequencing for variants in most likely candidate genes. They were then subjected to whole exome sequencing. Initial analysis was focused to identify variants within 250 genes associated with different forms of retinal dystrophies. Additional family members were also recruited to perform segregation analysis of the mutation with the disease phenotype. We present examples of cases that highlight the challenges and limitations of WES data analysis, which could have implication towards procedures used to identify mutations in gene diagnostics and research projects.

## Materials and Methods

### Ethics Statement

The study was conducted in accordance to the Helsinki Declaration and carried out according to the approved protocols at University of Zürich as per Swissmedic guidelines. The approval for genetic testing in the frame of this study was awarded to the Institute of Medical Molecular Genetics by the Federal Office of Public Health (FOPH) in Switzerland.

### Patients and families

Patients and family members were referred to us for genetic testing purposes from different eye clinics. All patients or family members as well as parents of affected children provided written informed consent for genetic testing. Pedigrees were drawn using PED6 software (http://www.medgen.de/ped/index.html). Information regarding family history, visual complaints and inheritance patterns of the diseases were collected through a standard ophthalmologic examination. All family members with a 5-digit patient ID represented in the pedigrees were included in this study. Family members not marked with a 5-digit ID did not participate in this study and no samples were analyzed.

### DNA extraction

Venous blood extracted from patients was used to isolate genomic DNA in duplicate using a coated magnetic bead technology according to the manufacturer’s recommendations (PerkinElmer Chemagen Technologie GmbH, Baesweiler, Germany). DNA integrity was verified using the Nanodrop (Life technologies, Darmstadt, Germany).

### Whole exome sequencing (WES) analysis

WES was performed at the Cologne Center for Genomics, University of Cologne, using NimbleGen SeqCap EZ Human Exome Library (Roche NimbleGen Inc., Madison, WI) for library preparation. Paired-end 100nt sequencing was performed on Illumina HiSeq2000. Alignment of sequence reads, indexing of the reference genome, variant calling and annotation was achieved using a pipeline based on BWA[5], Samtools[6], Picard (http://broadinstitute.github.io/picard/) and Annovar[7] respectively. Variants were annotated using Alamut-HT (Interactive Biosoftware, Rouen, France) and visualized on Alamut Viewer 2.2 (Interactive Biosoftware, Rouen, France). A filtering pipeline was established to remove known and frequent SNPs or benign polymorphisms. Variants with frequency less than 1% in the population were selected. Variants that have been described in literature and the Human Gene Mutation Database (HGMD) to be disease-associated were given higher priority. Within the variant types, protein truncation mutations leading to loss of function such as nonsense or frameshift mutations were given higher priority. Pathogenicity of missense variants were checked by five protein prediction algorithms SIFT[8], PolyPhen2[9], MutationTaster2[10], MAPP[11] and Align GVGD[12, 13].

### Primer design, PCR amplification and Sanger sequencing

Most likely disease-causing variants were confirmed by Sanger sequencing in the patient and the available family members. Primers were designed using Primer3 algorithm[14] and purchased at Microsynth AG (Balgach, Switzerland). All target regions were amplified in duplicate from genomic DNA of the patients and available family members using Hot FirePol^®^ DNA Polymerase (Solis BioDyne, Tartu, Estonia). PCR products were purified by treating them with ExoSAP reagent (Affymetrix, Santa Clara, CA) and sequenced using the Big Dye Terminator Cycle v1.1 Sequencing Kit (Applied Biosystems, Carlsbad, California, USA) and ABI Prism 3730 Genetic Analyzer (Applied Biosystems, Carlsbad, California, USA). Sanger sequencing data analysis was performed using the Sequencing Analysis Software v5.4, SeqScape v2.6 (Applied Biosystems, Carlsbad, California, USA), MutationSurveyorV5.0.0 (Soft Genetics, Pennsylvania, USA) and Chromas (Technelysium, South Brisbane, Australia) to identify the likely disease causing mutations. Mutation is defined as previously described[15].

### Cell culture and splicing assay

Patient derived fibroblasts were established as previously described[16, 17]. Cells were cultured in MEM medium substituted with 10% fetal calf serum, 1.3% L-glutamine, 0.8% antibiotic and antimycotic solution and incubated at 37°C and 5% CO_2_. 80% confluent cells were treated with 100μg/ml cycloheximide and incubated for 4 hours, upon which cells were harvested and total RNA was extracted using Qiagen RNeasy mini kit (Hombrechtikon, Switzerland). cDNA was generated from total RNA by reverse transcription with random primers according to manufacturer’s instructions (Supercript III, Invitrogen, Basel, Switzerland). Primers overlapping the intronic region (Intron 5 of *CDHR1* gene) were designed in order to amplify a specific product in case of activation of the cryptic splice site in the patient cell line. RT-PCR reaction was performed and PCR products were analyzed on agarose gel. RT-PCR products were verified by sequencing.

### Structural analysis

Structural modeling of CDHR1 (reference sequence NP_149091.1), PRPF31 (reference sequence NP_056444.3) and the respective mutant protein sequences was performed at iTasser server[18]. Visualization of the generated structures and their alignments were performed using PyMol (The PyMOL Molecular Graphics System, Version 1.8 Schrödinger, LLC).

## Results

Out of 11 cases diagnosed with either RP or CORD, we identified putative disease-associated variants in 8 cases. The clinical phenotypes of the patients, variant descriptions and pathogenicity predictions of these sequence variations are shown in Table 1.

**Table 1:** Clinical phenotypes of patient, variant descriptions and pathogenicity prediction of the variants identified in this study.

S. No.	Case ID	Origin of patient	Diagnosis	Gene	OMIM	Disease-Causing Mutation	Exon/ Intron	ExAC AltFreq_All	Zygosity	SIFT	PolyPhen2	MutationTaster2	MAPP	Align GVGD Class	Reference
1	27485	Switzerland	adRP	*RHO*	136880	NM_000539.3:c.170T>G:p.Leu57Arg	Ex 1	-	Heterozygous	Deleterious	Probably Damaging (0.991)	Disease causing	Bad	C0	Sullivan (2006) Invest Ophthalmol Vis Sci 47: 3052
2	23880	Switzerland	adRP	*PRPF8*	600059	NM_006445.3:c.7000dup:p.Tyr2334Leufs*51	Ex 43	-	Heterozygous	NA	NA	NA	NA	NA	This study
3	27536	Turkey	arRP DD: LCA	*RPGRIP1*	613826	NM_020366.3:c.2890del:p.Ser964Profs*37	Ex 17	-	Homozygous	NA	NA	NA	NA	NA	This study
4	24718	Switzerland	arCRD	*BEST1*	611809	NM_001139443.1:c.242G>A:p.Arg81His	Ex 3	0.00012	Homozygous	Deleterious	Probably Damaging (1.0)	Disease causing	Good	C0	Krämer (2000) Eur J Hum Genet 8: 286
5	26165	Switzerland	adRP	*RP1*	180100	NM_006269.1:c.2613dup:p.R872Tfs*2	Ex 4	-	Heterozygous	NA	NA	NA	NA	NA	Payne (2000) Invest Ophthalmol Vis Sci 41: 4069
6	25900	Switzerland	adRP	*RP1*	180100	NM_006269.1:c.2613dup:p.R872Tfs*2	Ex 4	-	Heterozygous	NA	NA	NA	NA	NA	Payne (2000) Invest Ophthalmol Vis Sci 41: 4069
7	26007	Switzerland	arRP	*CDHR1*	613660	NM_033100.2:c.398C>G:p.Pro133Arg& NM_033100.2:c.439-17G>A	Ex 5 & Int 5	- & 0.00002	Compound heterozygous	Deleterious & NA	Probably Damaging (1.0) & NA	Disease causing & NA	Bad & NA	C0 & NA	This study
8	23530	Switzerland	adRP	*PRPF31*	600138	NM_015629.3:c.548_580dup:p.Glu183_Met193dup	Ex 7	-	Heterozygous	NA	NA	NA	NA	NA	This study
9	22538	Switzerland	arCRD	-	-	Not yet identified	-	-	-	-	-	-	-	-	-
10	26309	Switzerland	adRP	-	-	Not yet identified	-	-	-	-	-	-	-	-	-
11	23609	Switzerland	arRP	-	-	Not yet identified	-	-	-	-	-	-	-	-	-

All identified variants described in this study (except *RP1* and *BEST1*variants) were absent in an in-house database of 130 exomes (260 alleles). *RP1* variant (NM_006269.1:c.2613dup) was identified in four additional families diagnosed with retinitis pigmentosa. *BEST1* variant (NM_001139443.1:c.242G>A) was heterozygous in an individual not known to be affected by retinal disease (allele frequency = 0.38% in our in-house database).

### WES analyses

**Fig 1:**
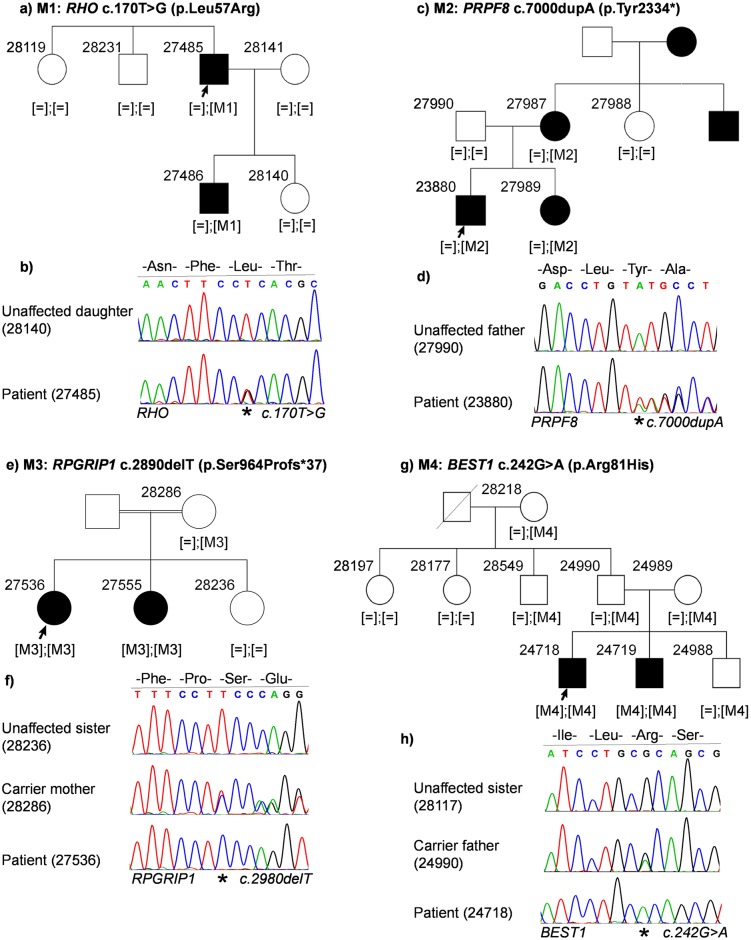
Patient pedigrees and sequence chromatography of identified disease-associated variants. Affected individuals are indicated with filled symbols and unaffected individuals are indicated with open symbols. Index patients are indicated with an arrow and were subjected to WES. Variants are denoted as M1, M2, M3 and M4 and zygosity of variants are indicated in third brackets below every analyzed family member. Sequence chromatography images of patients and representative family members are shown below the reference sequence. Mutated position on the chromatograph is depicted with an asterix. **(a)** Family pedigree of patient 27485. **(b)** Sequence chromatography of identified heterozygous *RHO* variant in patient (bottom) and unaffected daughter (top). **(c)** Family pedigree of patient 23880. **(d)** Sequence chromatography of identified heterozygous *PRPF8* variant in the patient (bottom) and unaffected father (top). **(e)** Family pedigree of patient 27536. **(f)** Sequence chromatography of identified homozygous *RPGRIP1* variant in the patient (bottom), heterozygous unaffected mother (middle) and unaffected sister (top). **(g)** Family pedigree of patient 24718. **(h)** Sequence chromatography of identified homozygous *BEST1* variant in patient (bottom), unaffected heterozygous father (middle) and unaffected sister (top).

Case ID 27485 (RP, autosomal dominant): The index patient is a 63-year old male patient diagnosed with RP. The family presented an autosomal dominant mode of inheritance with the son of the index patient also being affected. We identified a heterozygous missense mutation in *RHO*: NM_000539.3:c.170T>G:p.Leu57Arg (Fig 1a and 1b). This mutation was previously described[19] (HGMD_PRO = CM063096) and cosegregated with the disease in the family. The mutation is predicted to be damaging by four protein prediction algorithms (Table 1) and is located in the transmembrane domain.

Case ID 23880 (RP, autosomal dominant): The index patient is a 35-year old male with an affected sibling. His mother was also affected by RP but not his father. There were additional affected members in the maternal branch of the family, clearly pointing towards an autosomal dominant inheritance pattern. A novel heterozygous frameshift duplication was identified in *PRPF8* in the penultimate codon: NM_006445.3:c.7000dupA:p.Tyr2334* (Fig 1c and 1d). This variant was identified in all affected family members (patient, his sister and father). Unaffected family members did not carry this variant.

Case ID 27536 (RP, autosomal recessive; differential diagnosis: Leber congenital amaurosis, LCA): The patient is a 32-year old female of consanguineous parents who are not affected. She has one affected and one unaffected sister. A novel homozygous frameshift deletion was identified in exon 17 (out of 24 exons) of *RPGRIP1* in the patient and the affected sister: NM_020366.3:c.2890delT:p.Ser964Profs*37 (Fig 1e and 1f). This mutation leads to a frameshift and a premature stop codon 37 triplets downstream from the mutation. NGS variant calling initially suggested a heterozygous mutation in *RPGRIP1*. The mutation was excluded in the unaffected sister and the mother. Samples from the father of the patient were not available, but it can be assumed that, like the mother, he also is a carrier for this mutation.

Case ID 24718 (Cone-rod dystrophy, autosomal recessive): A homozygous missense mutation in *BEST1* was identified in the affected index patient (24718) and his affected brother (24719): NM_001139443.1:c.242G>A:p.Arg81His. They inherited one mutant allele from each parent; both heterozygous carriers for the mutation (Fig 1g and 1h). All other family members are unaffected and do not show any bestrophin-associated manifestations. They are either heterozygous carriers (28218, 28549, 24990 and 24989) or non-carriers (28197 and 28177) of the mutation. This mutation has previously been reported as disease-causing[20–22] (HGMD_PRO = CM001382). It is predicted to be deleterious (SIFT), probably damaging (Polyphen2 score = 1.0) and disease-causing (MutationTaster2).

**Fig 2:**
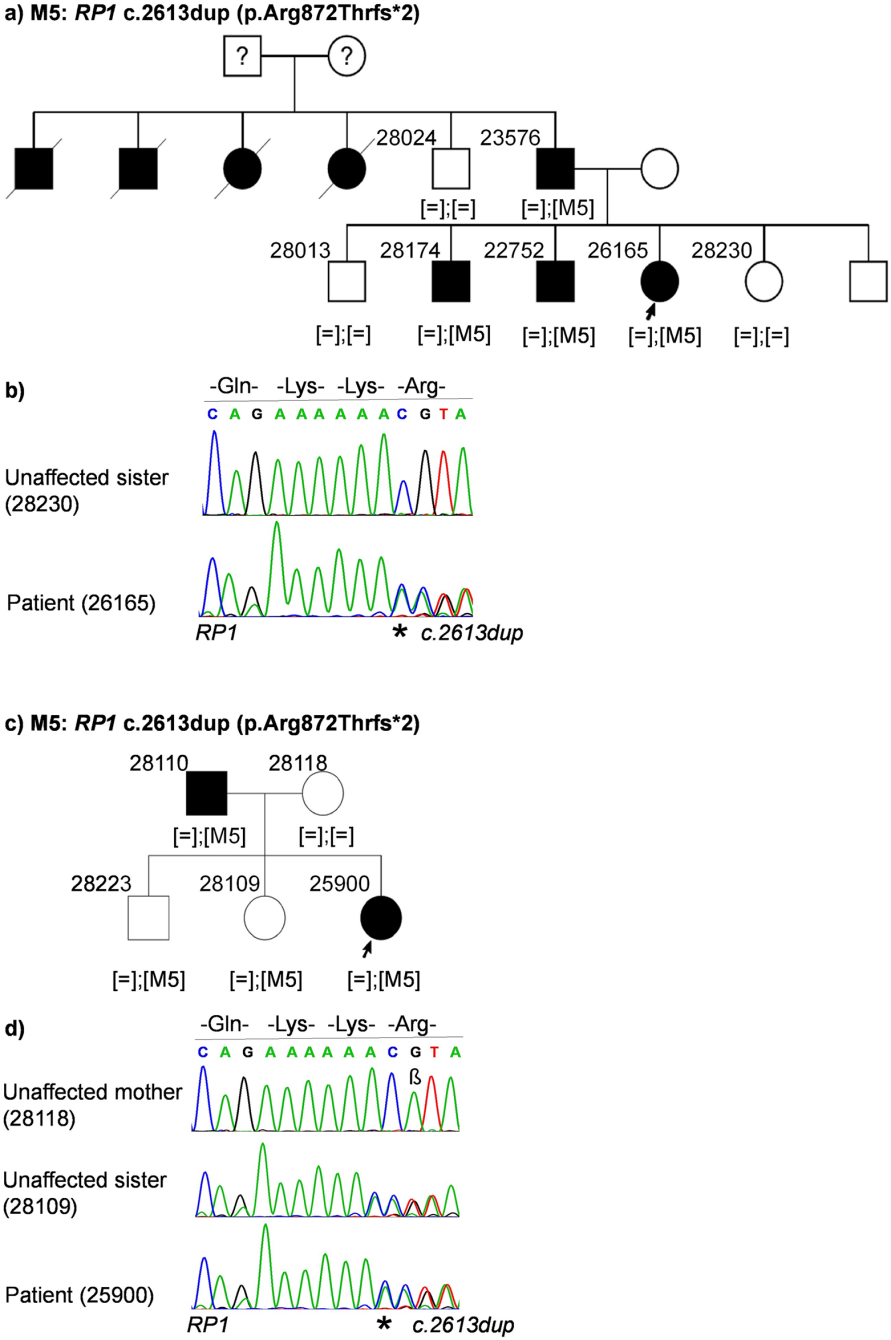
Patient pedigrees and sequence chromatography of identified disease-associated variants. Variants are denoted as M5. **(a)** Family pedigree of patient 26165. **(b)** Sequence chromatography of identified heterozygous *RP1* variant in patient (bottom) and unaffected sister (top). **(c)** Family pedigree of patient 25900. **(d)** Sequence chromatography of identified heterozygous *RP1* variant in patient (bottom), unaffected sister carrying the variant (middle) and unaffected mother (top). β: A known polymorphism at position c.2618 in *RP1* gene with a frequency of 26.98% in Europeans (Source: Exome Aggregation Consortium).

Case ID 26165 (RP, autosomal dominant): The index patient is a 54-year old female with two affected brothers, 3 unaffected siblings, an affected father and unaffected mother. The pedigree indicates an autosomal dominant inheritance pattern (Fig 2a). A heterozygous frameshift insertion was found in exon 4 in *RP1* (last exon): NM_006269.1:c.2613dup:p.Arg872Thrfs*2 (Fig 2b). This frameshift leads to a premature stop codon, 2 triplets downstream from the mutation. The mutation co-segregated with the disease within the family (Fig 2a). It has previously been described as disease-associated[23] (HGMD_PRO = CI004598). The mutation was absent in an ethnically matched control cohort representing 576 autosomal alleles.

Case ID 25900 (RP, autosomal dominant): The index patient is a 47-year old female with an affected father. Her mother and two siblings are unaffected (Fig 2c). The same frameshift insertion in *RP1* as described above was found in the patient and the affected father: NM_006269.1:c.2613dup:p.Arg872Thrfs*2. The mutation was also identified in two additional members in this family (28223 and 28109) who did not present any symptoms of the disease at the first clinical examination at the age of 37 and 40 years, respectively (Fig 2c and 2d).

**Fig 3:**
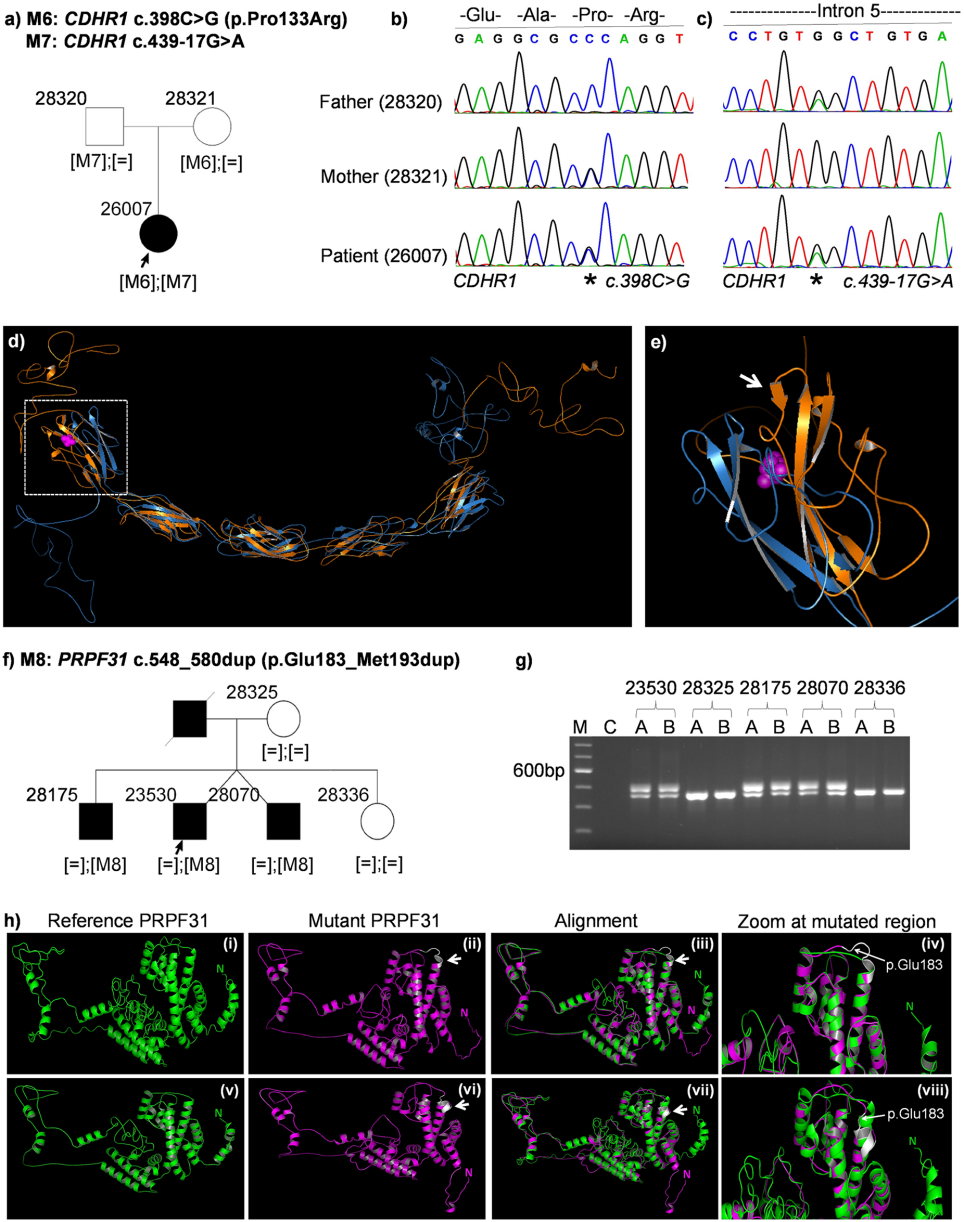
Patient pedigrees and sequence chromatography of identified disease-associated variants. Variants are denoted as M6, M7 and M8. **(a)** Family pedigree of patient 26007. **(b)** Sequence chromatography of heterozygous *CDHR1* variant c.398C>G in patient (bottom), carrier mother (middle) and father (top). **(c)** Sequence chromatography of heterozygous *CDHR1* variant c.439-17G>A in patient (bottom), mother (middle) and carrier father (top). **(d)** Predicted structure of CDHR1 reference protein (in blue) aligned to mutant CDHR1 (p.Pro133Arg) (in orange). The mutation is shown by magenta spheres and is localized within the first cadherin domain (white rectangle). **(e)** A zoomed image of the first cadherin domain of CDHR1 shows an additional beta-sheet (white arrow) close to the mutation **(f)** Family pedigree of patient 23530. **(g)** Agarose gel image of *PRPF31* exon 7 PCR shows a larger band only in affected members indicating a duplication. C = Water control in PCR. **(h)** Comparison of predicted models of the PRPF31 reference protein sequence (i & v, in green), mutant PRPF31 (ii & vi, in magenta), alignment of reference and mutant PRPF31(iii & viii) and zoomed image of the alignment at the mutation site (iv & viii). An additional turn of the mutant in the coiled-coil domain is depicted in white. The first amino acid of the 11bp duplication is shown by a white arrow. “N” denotes the N-terminus of the protein.

**Fig 4:**
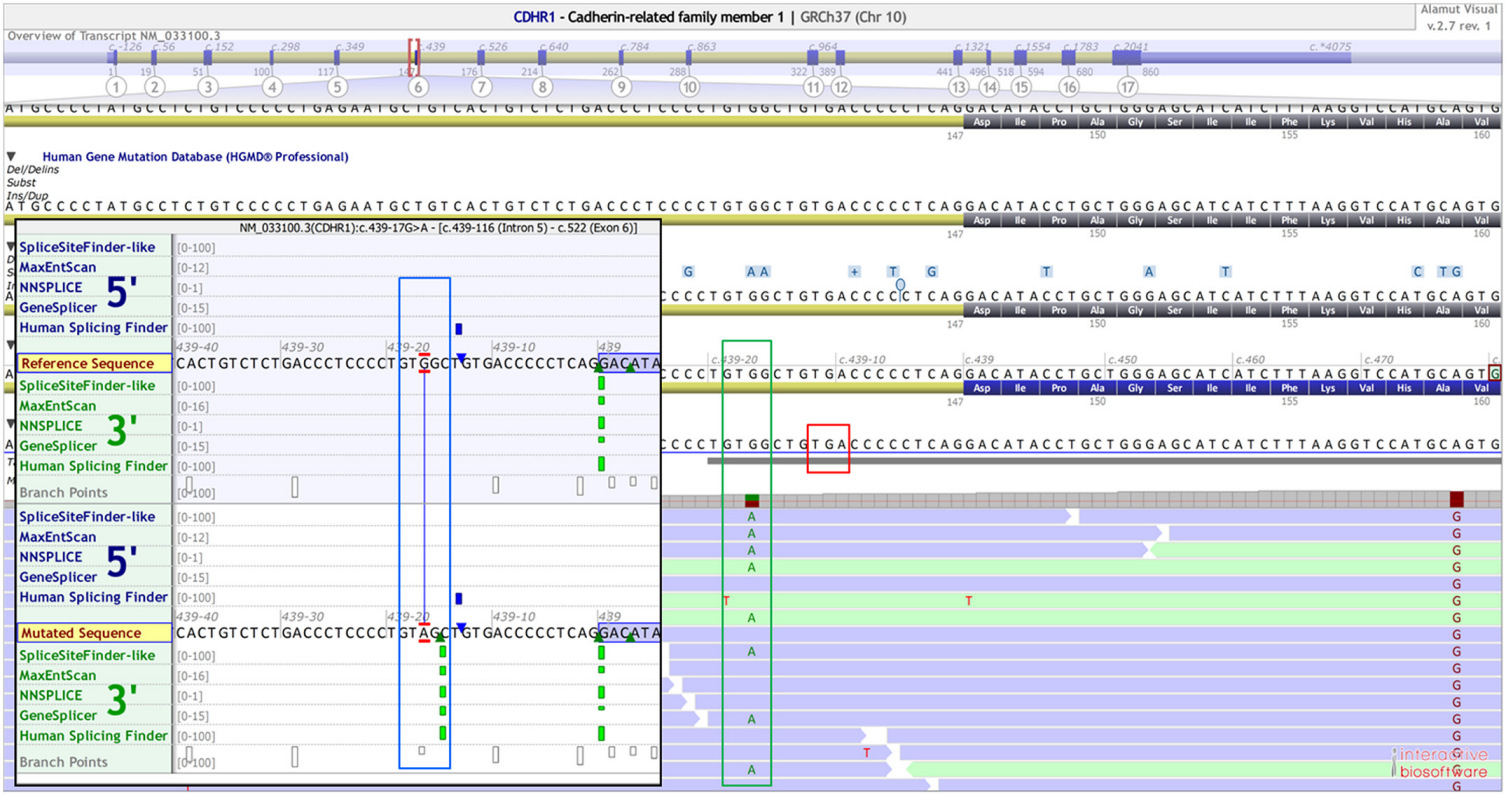
*CDHR1* intronic variant. Snapshot of Alamut visual showing an intronic variant (c.439-17G>A) in patient 26007 (green rectangle). The bam alignment file clearly shows a heterozygous variant 17bp upstream from exon 6. Inset: Snapshot of splicing prediction algorithms (*in silico*) shows a strong cryptic splice acceptor gain at the site of the variant (blue rectangle). Values are comparable to that of canonical splice acceptor site. Red rectangle shows a stop codon in-frame to the cryptic splice activator site.

Case ID 26007 (RP, autosomal recessive): The patient is a 59-year old woman clinically diagnosed with RP. Her parents were not affected (Fig 3a). We found novel, compound heterozygous mutations in *CDHR1*, encoding the cadherin-related family member 1, in the DNA of the patient. She inherited one mutant allele from each parent (Fig 3b and 3c). (i) NM_033100.2:c.398C>G:p.Pro133Arg (Missense) was maternally transmitted. It leads to the change of a highly conserved Proline to Arginine (S1 Fig, **red rectangle**). It is predicted to be deleterious (SIFT) and disease-causing (MutationTaster2). Structural prediction using iTasser showed that this missense variant leads to the creation of a short beta-sheet in the mutant protein which lies in the first cadherin domain of CDHR1 (Fig 3d and 3e). (ii) NM_033100.2:c.439-17G>A was inherited from her father. This variant is located 17 bases upstream of exon 6 and predicted to generate a cryptic splice acceptor site in intron 5 (Fig 4, **green rectangle**). The prediction values of the new splice acceptor using five different splice prediction algorithms (SpliceSite Finder-like, MaxEntScan, NNSPLICE, GeneSplicer and Human Splicing Finder) are comparable to that of the canonical splice acceptor site beginning at the conserved splice acceptor of exon 6 (Fig 4 **inset: blue rectangle**, Table 2). It seems likely that this new splice acceptor site is used by the splicing machinery in addition to the canonical site. This leads to a 15bp longer exon and generate a stop codon 3-basepairs downstream of the cryptic splice acceptor site (Fig 4, **red rectangle**). Both variants were absent in an ethnically matched control cohort of 576 alleles.

**Table 2:** Prediction scores of canonical (c.439) and cryptic splice site (c.439-17) by five prediction algorithms due to the variant c.439-17G>A in *CDHR1* gene.

		Canonical acceptor site (c.439)	Cryptic acceptor site (c.439-17)
	**SpliceSiteFinder-like**	80.5	Not predicted
	**MaxEntScan**	8.2	Not predicted
**Reference**	**NNSPLICE**	0.7	Not predicted
	**GeneSplicer**	5.7	Not predicted
	**Human Splicing Finder**	89.6	Not predicted
	**SpliceSiteFinder-like**	80.5	71.8
	**MaxEntScan**	6.5	9.2
**Mutant**	**NNSPLICE**	0.8	0.7
	**GeneSplicer**	3.8	8.1
	**Human Splicing Finder**	89.6	83.2

**Fig 5:**
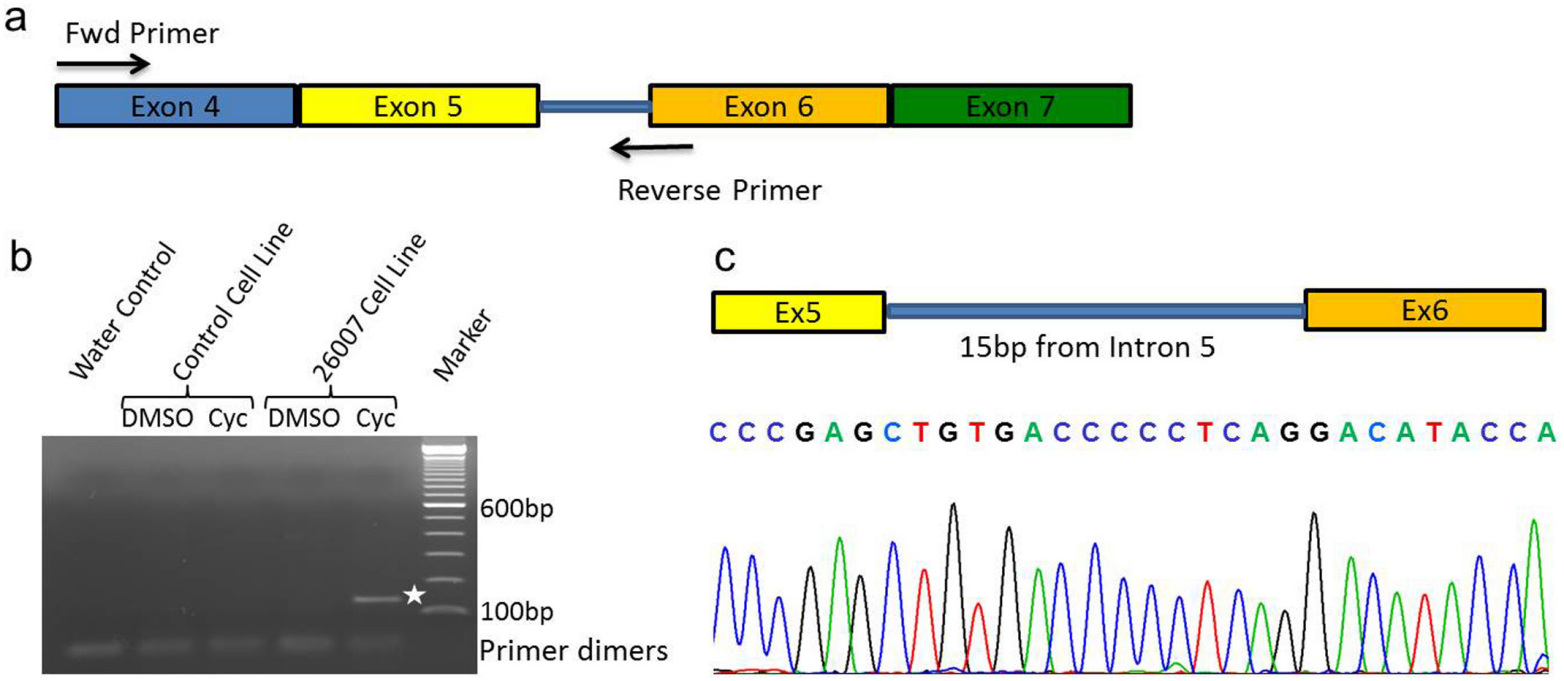
Analysis of alternative splicing *in vitro*. **(a)** Primer design to capture putative intron retention due to c.439-17G>A variant in *CDHR1*. **(b)** Agarose gel electrophoresis of RT-PCR showing a 140bp PCR product only in the patient cell-line (white asterix). No products were seen in control cell line or water control. **(c)** Bottom: Sequence chromatograph shows a clear retention of 15bp from intron 5 in the RT-PCR product, generated due to the cryptic splice-site activation. Top: Schematic representing the exon-intron boundaries as observed in the RT-PCR product.

Since the splice site is followed by a stop codon, we reasoned that the mutant transcript might be affected by nonsense-mediated mRNA decay. To test this hypothesis, we treated fibroblasts established from a skin biopsy of the patient in comparison to control fibroblasts (not carrying these mutations in *CDHR1*) with cycloheximide and DMSO as control. Subsequently, we isolated total RNA from these cell lines and performed RT-PCR. Upon gel electrophoresis, we identified an aberrant RT-PCR product (Fig 5b, white asterix) in the patient cell line treated with cycloheximide. The expected size of this product was 140bp. No product was amplified in the control cell line after DMSO or cycloheximide treatment, and in the patient cell line treated with DMSO (Fig 5b). Upon sequencing this PCR product, we confirmed that the predicted splice site activation occurred in this patient. It included a 15bp insert from intron 5 of *CDHR1* which corresponds to the predicted cryptic splice activation occurring due to the mutation c.439-17G>A (Figs 5c and 4 **inset**).

Case ID 23530 (RP, autosomal dominant): The index patient is a 43-year old male with an affected twin brother, a second affected brother, an unaffected sister and unaffected mother (Fig 3f). The father was reported to be affected. Among the known RP genes, no mutation explaining the disease was identified by using WES in this patient. However, upon screening of candidate genes by Sanger sequencing, a novel 33 base-pair duplication was found in the *PRPF31* gene (NM_015629.3:c.548_580dup: p.Glu183_Met193dup), which cosegregated with the disease in the family. The duplication was observed only in affected family members as seen by a larger PCR product on the agarose gel (Fig 3g). Structural analysis of the PRPF31 mutant protein aligned to the reference protein structure predicts the generation of an additional turn due to this 11 amino acid duplication in the second coiled-coil alpha helix domain of the PRPF31 protein (Fig 3h). The model with the highest confidence score (c-score) predicted that the additional turn was in continuation with the existing alpha helix (Fig 3hi–3hiv). A second model suggested that this additional helix turn looped out of the coiled coil domain (Fig 3hv–3hviii). In both models, the mutation is predicted to lead to loss of the N-terminal alpha-helix (Fig 3h, denoted by the letter N in green for the reference PRPF31 and magenta for the mutant PRPF31).

**Fig 6:**
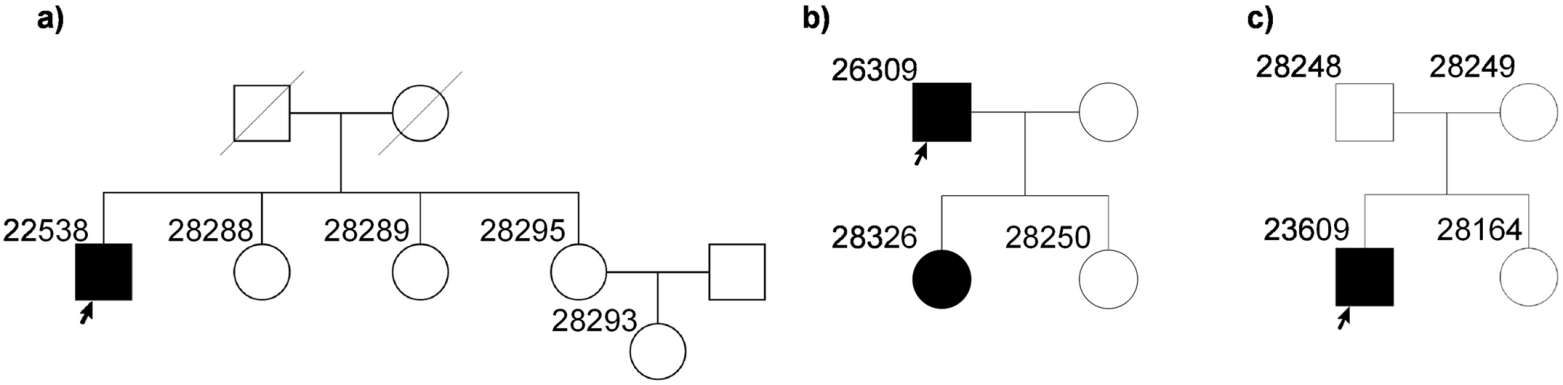
Pedigrees of families where underlying genetic mutations were not identified. (a) Family pedigree of patient 22538 diagnosed with CORD. (b) Family pedigree of patient 26309 diagnosed with RP. (c) Family pedigree of patient 23609 diagnosed with CORD.

In three families (one with a putative dominant mode of inheritance and two potentially recessive cases), we were not able to detect mutations in genes currently associated with IRDs, which could explain the disease phenotype based on the implicated mode of inheritance (Fig 6). These cases are being further investigated for identification of novel disease associated gene mutations.

## Discussion

IRDs show an extraordinary genetic and phenotypic heterogeneity. These diseases of the retina are currently associated with mutations in over 250 genes. With the advent of next generation sequencing (NGS), identification of the underlying genetic basis of IRDs has been revolutionized. Genetic diagnostics by means of panel or whole exome sequencing has been shown to be a reliable and fast method with a reported diagnostic efficiency between 50–65%[15, 24–27] (Tiwari et al, unpublished data). Although this number is higher compared to conventional Sanger sequencing and candidate gene approaches, a considerable percentage of cases still remains undiagnosed. Those undiagnosed patients are less frequent for syndromic cases[15] and in cases within consanguineous populations, where autozygosity-guided mutation analysis in combination with WES/NGS was shown to reveal higher diagnostic efficiency[28, 29]. We employed the well-established method of patient sample screening for mutations in most likely candidate genes and confirmed the identified variants in additional family members by segregation analysis. Some of the patients were prescreened for mutations in likely candidate genes (*RHO*, *PRPH2*, *RP2*, *RPGR*, *CRX*, NRL) using Sanger sequencing. In this study, which included one consanguineous family, we were able to identify disease-causing variants in 7 out of 11 cases using WES. A disease-associated variant in an additional case was identified only by Sanger sequencing. Our cohort consisted of 8 RP cases and 3 CORD cases, seven and one of whom revealed positive findings in this study, respectively. We identified 3 previously described mutations in 4 families and 5 novel variants in 4 families. The variant types included 3 frameshift, 3 missense and 1 splice mutation as well as 1 duplication of 33bp.

Although it has led to a higher diagnostic success rate, carefulness is needed during NGS data analysis, especially in diseases with a remarkable genetic heterogeneity, such as IRDs. The identification of disease-associated variants sometimes is very straightforward as in case 27485 (Fig 1a), where a known missense mutation in *RHO* was identified in the affected family members. Mutations in *RHO* account for the majority of autosomal dominant RP cases. It can be slightly more difficult when very few mutations have been identified in a gene. For example in case 23880 (Fig 1c), in whom a novel, single nucleotide duplication in the penultimate codon of *PRPF8* was identified. Mutations in *PRPF8* are one of the least frequent causes of dominant RP and therefore this gene is less likely to be screened by Sanger sequencing in traditional diagnostic laboratory screening pipeline. Amino acids 2301–2335 at the C-terminus of PRPF8 are known to interact with EFTUD2 and SNRNP200[30–32] and many mutations leading to RP have been shown to cluster at the C-terminal domain of PRPF8[30, 33]. An inefficient repression of SNRNP200 due to mutations in the C-terminus of PRPF8 has been hypothesized to be associated with PRPF8-linked RP[34]. The nonsense mutation p.Tyr2334* in PRPF8 in patient 23880 generates a premature termination codon in the protein. However, being in the penultimate amino acid, loss of function cannot be explained by nonsense mediated decay. Most likely, it leads to an inefficient repression of SNRNP200 function or loss of interaction with either EFTUD2 or SNRNP200.

If a panel approach is being used, the analysis is restricted to genes associated with a specific disease. For example in case 27536 (Fig 1e), the primary clinical diagnosis was RP with a differential clinical diagnosis of Leber congenital amaurosis (LCA). A RP panel will typically exclude *RPGRIP1*, because majority of the mutations described in *RPGRIP1* have been associated with LCA (n = 77, source: HGMD), while very few with RP (n = 4, source: HGMD), thus leading to an unsuccessful genetic diagnosis. In this case we identified a novel frameshift deletion in all affected family members in exon 17 (out of 24) of *RPGRIP1* (p.Ser964Profs*37), which leads to a premature termination codon 37 triplets downstream from the mutation and most likely results in non-sense mediated decay of the transcript. Retrospectively, the differential diagnosis of LCA could more aptly describe the clinical phenotype in this patient. Similarly in case 24718, CORD is due to a previously described homozygous missense mutation in *BEST1* (p.Arg81His)[20]. Although the majority of *BEST1* mutations are dominantly inherited, mutations in *BEST1* are associated with both recessive and dominant forms of Best macular dystrophy[35]. *BEST1* is not the most likely candidate for CORD, and as such, *BEST1* would be excluded from a typical CORD diagnostic gene panel. Functional studies have shown that this mutation leads to reduced chloride conductance and increased proteasomal degradation of the BEST1 protein [21, 22, 36].

Analysis pipelines and variant-calling algorithms are being improved continuously and therefore chances of a wrong call can pose problems with analysis. For example in case 27536, zygosity of the *RPGRIP1* mutation c.2890delT was annotated to be heterozygous instead of homozygous. Since, the parents were consanguineous and unaffected, we expected in this case a homozygous or compound heterozygous variant(s) to be causative for the disease. Only upon a detailed reanalysis of this specific mutation, we identified that the sequence included 6 T-allele reads and 49 deletions of the T-allele, suggesting atypical allele balance (<20% reference allele) in combination with a strand bias of the T-allele (S2 Fig). Upon verification by Sanger, we could confirm this deletion to be homozygous.

Gene mutations, which could be either recessively or dominantly inherited, or show incomplete penetrance, are particularly challenging in making precise interpretation of the genetic data and results, an example being *RP1*. In families of patients 26165 and 25900, a previously described dominantly inherited frameshift duplication in *RP1* was identified[23] (Fig 2a–2d). In the family of patient 26165, all affected members were heterozygous for the mutation and non-affected family members did not carry the mutation. However, in the family of patient 25900, two unaffected siblings (one brother and one sister) were also heterozygous for the mutation (Fig 2c and 2d). This could be due to incomplete penetrance as dominant mutations in *RP1* have been shown to exhibit variable expressivity[37, 38]. A later age of onset of the disease in these family members could also explain this discrepancy. Therefore, a clinical re-examination would be required in these cases. We have also identified this mutation in four additional families with autosomal dominant RP in our cohort (data not shown), supporting the pathogenicity of the sequence variant.

CDHR1 belongs to the cadherin superfamily of calcium-dependent cell adhesion molecules. It encodes a photoreceptor-specific cadherin that plays a role in outer segment disc morphogenesis. It may be required for the structural integrity of the outer segment of photoreceptor cells and has been shown to interact with PROM1[39]. We identified compound heterozygous mutations in a trio where the index patient was affected with RP (Fig 3a). The missense variant in *CDHR1* (Pro133Arg) affects a highly conserved amino acid and is localized in the first cadherin domain of the protein. Structural analysis predicted a new beta-sheet in this cadherin domain due to the missense variant. Cadherins are involved in calcium-dependent cell-cell adhesion. Another proline substitution has been identified at the end of the fifth cadherin domain in a Spanish RP patient[40]. It can be hypothesized that the mutation perturbs the cell-cell adhesion role of the cadherin domain. Alternatively it could mediate its pathogenicity by a mechanism not yet described for CDHR1. The variant c.439-17G>A of *CDHR1* was predicted to activate a cryptic splice activator site *in silico*. Using fibroblasts from the patient, this activation was confirmed in a patient-derived cell line. Often, the focus of mutation identification lies within the coding regions. Splice changes affecting positions +/-2 and +5 base-pairs around exons are also given importance. However, padding regions could also harbor mutations which could lead to alternative splicing. These regions vary greatly between exons and depend upon the enrichment method being used. In addition, variants lying at the extremities of padding regions typically show strand bias and thus get excluded from many analysis pipelines. It is therefore important to carefully analyze the “padding” region of the exome capturing sequences, in order to not miss mutations lying further away from the canonical splice acceptor and donor sites. This also highlights the necessity of functional assays to validate predicted effects of variants e.g., missense or splice-site changes. In this case, only the transcript analysis of the patient-derived cell line confirmed the splice defect.

In patient 23530, none of the variants identified upon WES explain the dominant RP phenotype in the family. We included the patients’ DNA in a Sanger screening project of *PRPF31* gene, mutations in which account for the second highest number of disease-associated mutations in autosomal dominant RP cases. With Sanger sequencing, a novel 33bp duplication was identified in *PRPF31* in the patient and all affected family members. This case illustrates an important limitation of this NGS approach, where a large duplication was missed in WES data but could only be identified by conventional Sanger sequencing. Structural analysis predicts the generation of an additional turn in the alpha helix of the coiled-coil domain of the protein. It is likely that the mutation perturbs the interaction of PRPF31 with PRPF6[41]. Improved bioinformatics algorithms to detect duplications or deletions larger than 20 base pairs might help in identifying such mutations. However, such specific analyses are not routinely used and therefore it is important to consider using alternative analysis methods for cases still lacking a genetic diagnosis.

In conclusion, our study shows the power of WES in identifying pathogenic mutations in IRDs with a success rate of 63% but also its limitations. There might be multiple mutations per case in different genes, in addition to de-novo sequence variations that might not co-segregate with the disease[15]. Moreover, mutations located within and outside of the captured exonic regions should be carefully evaluated for effects on splicing or additional regulatory effects. In addition, a complementary approach to WES can be very helpful to identify IRD-associated mutations in cases lacking a genetic diagnosis.

## Supporting Information

S1 Fig Alamut screenshot of *CDHR1* variant NM_033100.2:c.398C>G (p.Pro133Arg). This variant affects an amino acid that is conserved from Tetraodon to humans (red rectangle).
(TIF)

S2 Fig Alamut screenshot of *RPGRIP1* variant NM_020366.3:c.2890delT (p.Ser964Profs*37). This 1bp deletion leading to frameshift was annotated as heterozygous. Values of the reads in inset show 49 deletions and 6 T-alleles (which shows a strand bias). Sanger sequencing confirmed this deletion to be homozygous.
(TIF)
