# Supplementary figures and images for: Identification of Novel and Recurrent Disease-Causing Mutations in Retinal Dystrophies Using Whole Exome Sequencing (WES): Benefits and Limitations
Source: PLoS One. 2016 Jul 8;11(7):e0158692. doi: 10.1371/journal.pone.0158692 (PMC4938416; doi:10.1371/journal.pone.0158692)

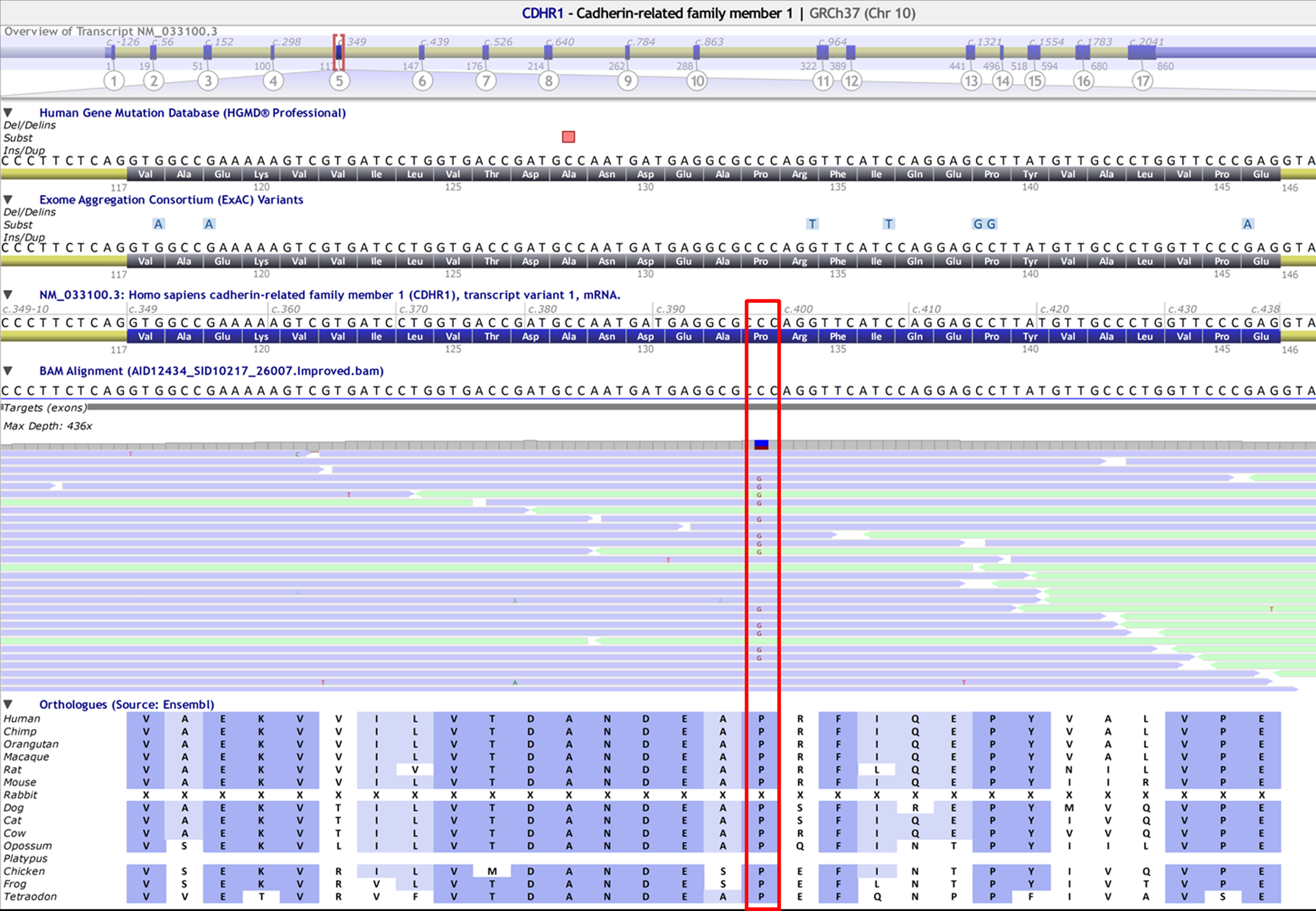

Supplement: S1 Fig — This variant affects an amino acid that is conserved from Tetraodon to humans (red rectangle). (TIF) [file pone.0158692.s001.tif]

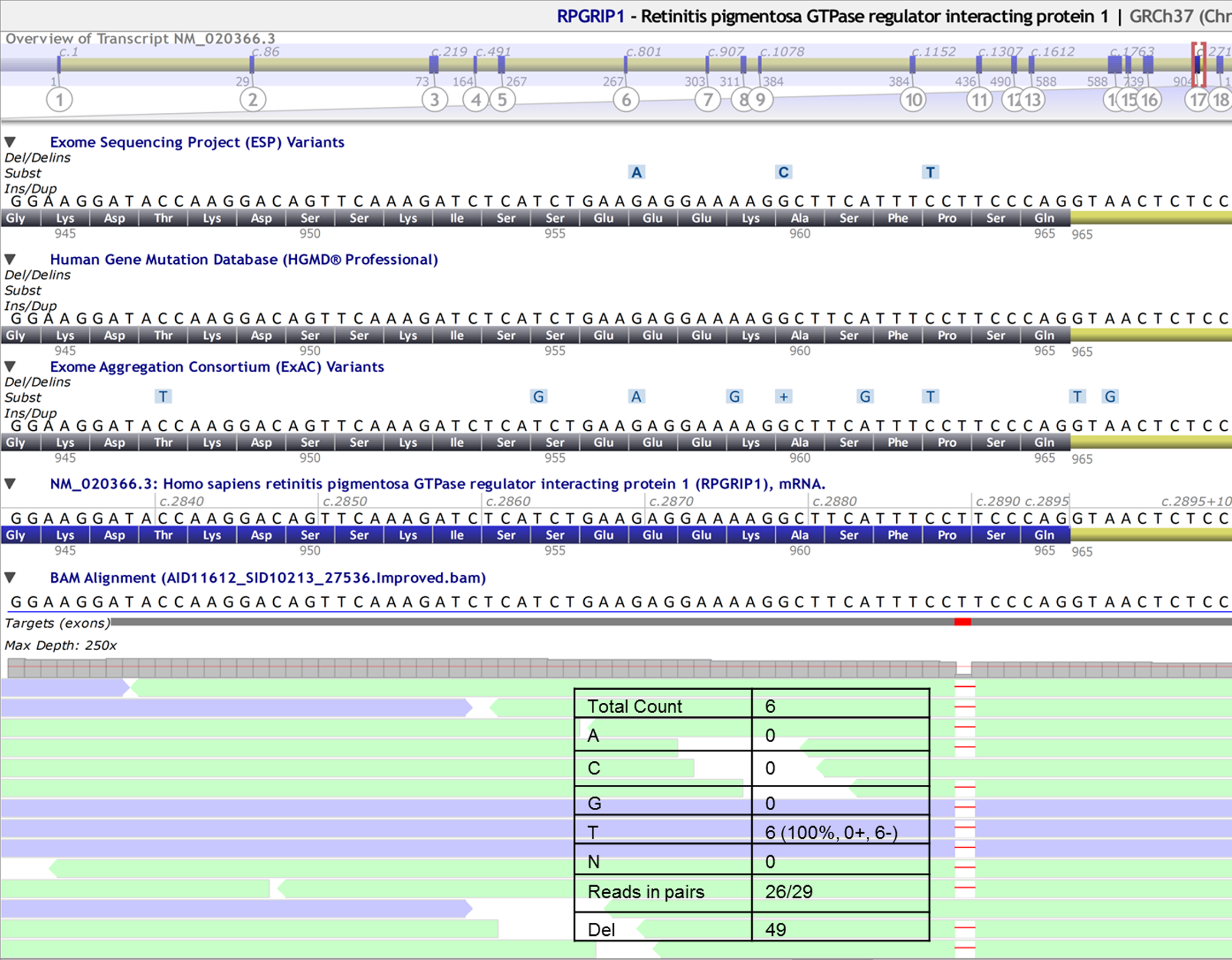

Supplement: S2 Fig — This 1bp deletion leading to frameshift was annotated as heterozygous. Values of the reads in inset show 49 deletions and 6 T-alleles (which shows a strand bias). Sanger sequencing confirmed this deletion to be homozygous. (TIF) [file pone.0158692.s002.tif]
